# Management of non-muscle-invasive bladder cancer: quality of clinical practice guidelines and variations in recommendations

**DOI:** 10.1186/s12885-019-6304-y

**Published:** 2019-11-06

**Authors:** Jing Zhang, Yunyun Wang, Hong Weng, Danqi Wang, Fei Han, Qiao Huang, Tong Deng, Xinghuan Wang, Yinghui Jin

**Affiliations:** 1 Department of Urology, Zhongnan Hospital of Wuhan University, No. 169, Donghu Road, Wuchang District, Wuhan, 430071 China; 2 Center for Evidence-Based and Translational Medicine, Zhongnan Hospital of Wuhan University, No. 169, Donghu Road, Wuchang District, Wuhan, 430071 China; 3 The First Clinical College of Wuhan University, Wuhan University, No. 99, Zhangzhidong Road, Wuchang District, Wuhan, 430072 China; 4 School of Basic Medical Sciences, Wuhan University, No. 115, Donghu Road, Wuchang District, Wuhan, 430071 China; 5 Emergency Department, Xuan Wu Traditional Chinese Medicine Hospital, No.8, Wanming Road, Xicheng District, Beijing, 10000 Beijing China

**Keywords:** Bladder cancer, NMIBC, Clinical practice guidelines, AGREE II, Management

## Abstract

**Background:**

Bladder cancer (BC) has become a major worldwide public health issue, especially non-muscle-invasive bladder cancer (NMIBC). A flood of related clinical practice guidelines (CPGs) have emerged; however, the quality and recommendations of the guidelines are controversial. We aimed to appraise the quality of the CPGs for NMIBC within the past 5 years and compare the similarities and differences between recommendations for therapies.

**Methods:**

A systematic search to identify CPGs for NMIBC was performed using electronic databases (including PubMed, Embase, Web of Science), guideline development organizations, and professional societies from January 12, 2014 to January 12, 2019. The Appraisal of Guidelines Research & Evaluation (AGREE) II instrument was used to evaluate the quality of the guidelines. Intraclass correlation coefficient (ICC) analysis was performed to assess the overall agreement among reviewers.

**Results:**

Nine CPGs were included. The overall agreement among reviewers was excellent. The interquartile range (IQR) of scores for each domain were as follows: scope and purpose 69.44% (35.42, 85.42%); stakeholder involvement 41.67% (30.56, 75.00%); rigour of development 48.96% (27.08, 65.63%); clarity and presentation 80.56% (75.00, 86.11%); applicability 34.38% (22.92, 40.63%) and editorial independence 70.83% (35.42, 85.42%). The NICE, AUA, EAU and CRHA/CPAM clinical practice guidelines consistently scored well in most domains.

It was generally accepted that the transurethral resection of bladder tumour (TURBT) and intravesical chemotherapy should be performed in the management of bladder cancer. The application of chemotherapy was highly controversial in high risk NMIBC. The courses of BCG maintenance were similar and included 3 years of therapy at full maintenance doses.

**Conclusions:**

The quality of NMIBC guidelines within the past 5 years varied, especially regarding stakeholders, rigour and applicability. Despite many similarities, the recommendations had some inconsistencies in the details.

## Background

Bladder cancer (BC), the 10th most common form of cancer worldwide, has become a major global public health issue [1]. Approximately 75% of BCs do not involve the muscle wall of the bladder [2]. Timely and effective treatment for non-muscle-invasive bladder cancer (NMIBC) can achieve good outcomes, potentially avoiding increase in recurrence rates and progression to muscle-invasive bladder cancer (MIBC) [3].

To optimize patient health care, the use of unnecessary medical intervention should be minimized, and cost-effectiveness should be improved. Clinical practice guidelines (CPGs) for NMIBC drafted by many national and international organizations have therefore been developed.

According to the Institute of Medicine (IOM), a trustworthy CPG is to “be developed via a transparent process by a group of multidisciplinary experts (including patient representatives) screened for minimal potential bias and conflicts of interest, and supported by a systematic review (SR) of the evidence” [4].

Given the standardization of the evidence-based medicine paradigm and concerns about the quality of care and increasing healthcare costs, the flood of CPGs for NMIBC has been accompanied by growing concerns about the variations in guideline recommendations and quality.

There has been considerable debate regarding the management of NMIBC, the clinical course of which is variable and complicated. Significant consensus exists in the majority of areas despite some variations in NMIBC guidelines [5].

To our knowledge, the quality of NMIBC guidelines has not yet been systematically searched and appraised. Therefore, to assist clinicians and patients in the field to make decisions about appropriate healthcare for specific clinical circumstances, we have thoroughly reviewed NMIBC guidelines published within the past 5 years, evaluated the quality of NMIBC guidelines, summarized the management of NMIBC and identified the discrepancies and consistencies.

## Methods

### Strategy for NMIBC guideline search

An exhaustive search (from January 12, 2014 to January 12, 2019) was performed in the PubMed, Embase, and Web of Science databases using a combination of text-free terms and their corresponding MeSH terms, as well as four major Chinese academic databases. The search strategy on PubMed is outlined in Additional file 1.

We also searched the websites of guideline development organizations and professional societies. A list of the websites with potential NMIBC guidelines are outlined in Additional file 2.

### Identification of guidelines for NMIBC

All guidelines related to NMIBC published in English or Chinese were included. A document was considered a guideline if it met the following criteria: (1) Explicit recommendations on the management of NMIBC have been provided. Only the CPGs including recommendations of transurethral resection of bladder tumour (TURBT) and intravesical therapy were included. (2) Evidence-based guidelines. To determine whether the guidelines were evidence-based, we investigated whether they reported a search strategy, literature quality or data extraction that classified the level of evidence (LOE) and graded the strength of recommendation (SOR). (3) Only the recent updated version was included. Single-author overviews, consensus statements, translations of CPGs and adapted CPGs were excluded.

### Evaluation of NMIBC guidelines

Four reviewers (J.Z., H.W., Y.Y.W. and Q.H.) from different backgrounds, consisting of urologists and methodologists, with extensive experience in evaluating CPGs independently evaluated the eligible guidelines using the AGREE II instrument. AGREE II consists of 23 key items organized within 6 domains (scope and purpose, stakeholder involvement, rigour of development, clarity and presentation, applicability, and editorial independence) [6].

Each domain identified a unique dimension of guideline quality rated on a 7-point scale scored from 1 (strongly disagree) to 7 (strongly agree). We summarized the domain scores individually and scaled the total of that domain, calculated by the following formula: (obtained score - minimal possible score)/(maximal possible score - minimal possible score) × 100% [6].

### Data collection

Two reviewers (T.D., D.Q.W.) independently extracted the details of the guidelines pertaining to the CPG characteristics, such as target disease, guideline developers, LOE and SOR of guidelines, and the related recommendations. The records of the two reviewers were compared, and any disagreement was resolved based on the evaluation of a third reviewer (F.H.).

Whereas various grading systems have been used to evaluate the LOE and SOR in different guidelines, for the convenience of statistics, we discussed and reached a consensus on a composite grading system generated in Additional file 3 for presenting the evidence and recommendations.

### Synthesis of guideline recommendations for NMIBC

We conducted a textual descriptive synthesis to analyse the scope, content, and consistency of the included recommendations related to the management of NMIBC. The synthesis was divided into the following sections and items: (1) TURBT and re-TURBT; (2) immediate postoperative instillation of intravesical chemotherapy; (3) measures to optimize chemotherapy administration; (4) induction and maintenance intravesical chemotherapy or immunotherapy; (5) side effects of and contraindication for Bacille Calmette-Guérin (BCG). Only recommendations with any assigned grade could be extracted.

### Data statistical analysis

A descriptive statistical analysis was performed by calculating each domain score and scaled domain score. The data for each AGREE II domain were provided as medians and interquartile ranges (IQRs).

Agreement among four reviewers was tested with intraclass correlation coefficient (ICC) with a 95% confidence interval (CI) for each domain. According to the scale proposed by Fleiss, the degree of agreement between 0.00 and 0.40 was deemed poor, 0.41 to 0.75 was fair to good, and 0.75 to 1.00 was excellent [7]. Statistical analyses were conducted using SPSS version 19.0 (SPSS Inc., Chicago, IL, USA).

## Results

**Fig. 1:**
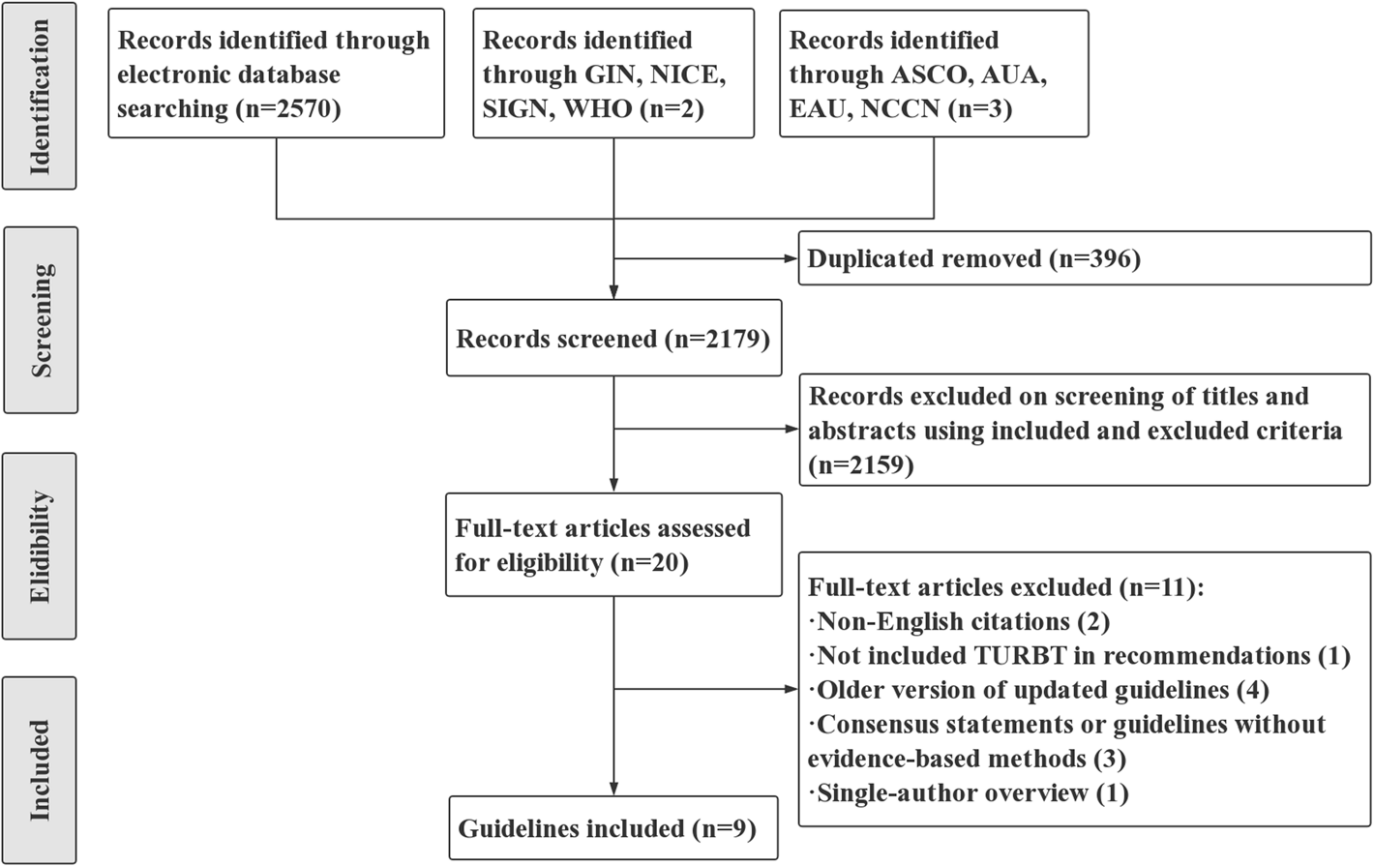
Flow chart of the identification process of CPGs for NMIBC

The flow chart in Fig. 1 shows the process by which we screened and selected the guidelines. Ultimately, there were 9 guidelines that met the inclusion criteria [3, 8–15]. For every guideline that was ultimately included, we systematically collected all accompanying technical and supporting materials to better inform our assessments [16, 17]. The characteristics of the eligible guidelines are listed in Table 1.

**Table 1:** Characteristics of the identified guidelines on management of NMIBC

Guideline ID	Target disease	Development organization	Version		Country	Funding	Pages
			The edition	The type			
ESMO, 2014 [8]	BC	European Society for Medical Oncology, ESMO	The second	Full version	Europe	Not reported	9
NICE, 2015 [9]	BC	National Institute for Health and Care Excellence, NICE	The first	Full version; NICE version; NICE pathways; Information for the Public (IFP)	U.K.	NICE	500
CUA, 2015 [10]	NMIBC	Canadian Cancer Society, CUA	The second	Full version	Canada	Not reported	15
AUA/SUO, 2016 [3]	NMIBC	American Urological Association, AUA/Society of Urologic Oncology, SUO	The first	Full version	America	AUA	45
JUA, 2016 [11]	BC	Japanese Urological Association, JUA	The second	Full version	Japan	JUA	6
EAU, 2018 [12]	NMIBC	European Association of Urology, EAU	Updated annually	Full version; Pocket guideline; Translated version	Europe	EAU	48
ICUD/SIU, 2018 [13]	NMIBC	International Consultation on Urological Diseases, ICUD/Société Internationale d’Urologie, SIU	The fifth	Full version	International	Not reported	10
CRHA/CPAM, 2018 [14]	NMIBC	Chinese Research Hospital Association, CRHA/China International Exchange and Promotive Association for Medical and Health Care, CPAM	The first	Simplified version	China	The National Key Research and Development Program of China; Health and Family Planning Commission of Hubei province joint funding project	6
NCCN, 2019 [15]	BC	National Comprehensive Cancer Network, NCCN	Updated every few months	Full version	America	Not reported	103

### Quality assessment of guidelines

The ICC values for appraisal of the identified guidelines ranged from 0.81 to 0.97, indicating a good agreement among appraisers. The overall quality of the included CPGs was moderate, with the domain ‘clarity of presentation’ receiving the highest score, and the domain ‘applicability’ receiving the lowest score (Table 2, Additional file 4).

**Table 2:** AGREE II domain scores of included CPGs for NMIBC

Guideline ID	Domain Score (%)					
	Scope and purpose	Stakeholder involvement	Rigour of development	Clarity of presentation	Applicability	Editorial independence
ESMO, 2014 [8]	29.17	22.22	19.27	75.00	34.38	35.42
NICE, 2015 [9]	86.11	97.22	76.04	91.67	81.25	87.50
CUA, 2015 [10]	54.17	27.78	44.79	75.00	22.92	33.33
AUA/SUO, 2016 [3]	69.44	43.06	55.21	79.17	22.92	75.00
JUA, 2016 [11]	69.44	41.67	27.08	81.94	22.92	70.83
EAU, 2018 [12]	76.39	75.00	65.63	93.06	44.79	85.42
ICUD/SIU, 2018 [13]	48.61	30.56	21.88	66.67	14.58	52.08
CRHA/CPAM, 2018 [14]	81.94	76.39	66.67	80.56	40.63	85.42
NCCN, 2019 [15]	56.94	41.67	48.96	86.11%	38.54	20.83
ICC (mean ± SD)	0.94 ± 0.05	0.97 ± 0.02	0.97 ± 0.02	0.81 ± 0.15	0.91 ± 0.07	0.91 ± 0.07
Median score (IQR)	69.44	41.67	48.96	80.56	34.38	70.83
	(54.17, 76.39)	(30.56, 75.00)	(27.08, 65.63)	(75.00, 86.11)	(22.92, 40.63)	(35.42, 85.42)

*IQR* Interquartile range

### Scope and purpose

Guidelines for this domain received a median score of 69.44% with the IQR ranging from 35.42 to 85.42%. The highest score in this domain was 86.11%, as the guideline clearly defined its scope and global objectives and specifically defined the related clinical field and target populations [9].

### Stakeholder involvement

The guidelines appraised received the second lowest scores for stakeholder involvement (median, 41.67%; IQR: 30.56 to 75.00%). Six guidelines (66.67%) scored lower than 50% for domain ‘stakeholder involvement’ [3, 8, 10, 11, 13, 15]. Another three guideline panels consisted of a multidisciplinary group of covering clinicians [9, 12, 14], methodologists [9, 12, 14], pharmacists [14] and administrative staff [14]. Two guidelines involved patients or their representatives in guideline development to consider the preferences of the target population [9, 14].

### Rigour of development

The median score for the domain ‘rigour of development’ was 48.96% with an IQR ranging from 27.08 to 65.63%. Five guidelines (55.56%) scored lower than 50% [8, 10, 11, 13, 15], this was probably because these guidelines did not report the systematic methods for searching or evaluating the evidence [8, 11, 13]. Only one guideline described the process of how final decisions were made [14]. The proportions of SRs in evidence types were approximately 11.27% [10], 12.78% [3], 14.39% [12] and 14.73% [9] in four guidelines that presented their body of evidence clearly.

### Clarity of presentation

The domain ‘clarity of presentation’ received the median score of 80.56% (IQR: 66.67–93.06%), with all guidelines scoring > 60%, as the most relevant recommendations in all guidelines could be easily found with explicit SOR and LOE.

### Applicability

The domain ‘applicability’ received the lowest median score (median 34.38%; IQR: 22.92 to 40.63%). In general, there was little information regarding potential organizational barriers, cost implications, and tools for application, except for the NICE guideline [9], which scored 81.25%. Some derivative products including pathways [9], summaries for the public [9], quick reference document [12] and various translation versions [12], could be useful for application. Cost effectiveness was considered only in the NICE guideline, which involved health economists in guideline panels, incorporated health economics evidence and discussed implications for budgets behind recommendations [9].

### Editorial independence

The greatest range of scores was observed in the domain ‘editorial independence’ (IQR: 35.42, 85.42%). Although all the guidelines disclosed their conflicts of interest (COI), the quality of disclosure was not ideal. They gave minimal information about ways in which any COI were managed in either tabular or narrative form. A complete summary of the process for identifying, managing and reporting COI during guideline development was only presented in one of the guidelines [14].

### Synthesis of recommendations

Of the 9 guidelines, one guideline did not present the LOE underpinning the recommendations [11], and the remaining eight guidelines used six grading systems to rate the LOE and seven grading systems to rate the SOR (Additional file 5).

A total of 177 recommendations on the management of NMIBC were extracted for statistics (Additional file 6). Three guidelines tended to formulate a recommendation supported by more than one type of evidence, resulting in no correspondence between the number of types of evidence and recommendations [9, 10, 12]. It could be clearly seen that recommendations rated as grade A (33.9%) plus grade B (49.7%) accounted for a higher proportion, whereas evidence rated as level 2 (48.1%) plus level 3 (20.9%) accounted for a higher proportion.

To demonstrate differences between the identified guidelines, the key recommendations for the management of NMIBC were extracted and summarized (Tables 3, 4 and 5, Additional files 7, 8 and 9). Although the contents of recommendations achieved a significant consensus in most areas, there were some noteworthy discrepancies in these guidelines.

**Table 3:** Recommendations of TURBT and re-TURBT^a^

Guideline ID	TURBT			Re-TURBT							
	Be recommended	Adequate resection with muscle in specimen		No muscle in original specimen		Ta		T1		Interval between TURBT and Re-TURBT	
	SOR/LOE	G&S	SOR/LOE	G&S	SOR/LOE	G&RS	SOR/LOE	G	SOR/LOE	Interval	SOR/LOE
ESMO, 2014 [8]	−/−	–	A/I	–	−/−	High-risk	B/II-III	–	B/II-III	–	−/−
NICE, 2015 [9]^b^	−/−	–	A/low-very low	–	A/low-very low	High-risk	A/low-very low	–	A/low-very low	6	A/low-very low
CUA, 2015 [10]	−/−	–	A/−	Only T1	A/−	HG	C/−	–	C/−	2–6	A/−
AUA/SUO, 2016 [3]	−/−	–	−/−	–	Strong/B	High-risk, HG	Moderate/C	–	Strong/B	6	−/−
JUA, 2016 [11]	A/−	–	−/−	–	−/−	–	−/−	HG	A/−	–	−/−
EAU, 2018 [12]	Strong/1b	Except for TaG1/LG	Strong/1b	Except for TaG1/LG and primary CIS	Strong/1b-3	–	−/−	–	Strong/1b-3	2–6	Weak/3
ICUD/SIU, 2018 [13]	C/3	–	−/−	–	B/2	HG	C/3	–	B/2	6	B-C/2–3
CRHA/CPAM, 2018 [14]	A/4	Except for TaG1/LG	A/4	–	A/4	G3/HG	A/4	–	A/4	6	A/4
NCCN, 2019 [15]^c^	−/−	Except for TaLG	B/2A	Only HG	B/2A	> 3 cm or multi-focal	B/2A	–	B/2A	6	B/2A

*G* Grading, *S* Staging, *RS* Risk stratification, *HG* High grade, *CIS* Carcinoma in situ

^a^ The SOR and LOE are presented as “SOR/LOE”. “-” indicates that the recommendation or evidence was not presented

^b^ To simplify the table, we used “A” and “B” instead of “should/should not/offer/do not offer/refer/advise” or “consider” for presenting SOR

^c^ To simplify the table, we used “A” and “B”, “C” instead of “preferred intervention”, “other recommended intervention”, or “useful in certain circumstances” for presenting SOR

**Table 4:** Recommendations of intravesical therapy for low and intermediate risk patients^a^

Guideline ID	Low risk	Intermediate risk									
	Induction	Induction				Maintenance					
	Not be recommended	Be recommended		Course of treatment		Be recommended		Course of treatment			
	SOR/LOE	Chemotherapy	BCG			Chemotherapy	BCG	Chemotherapy		BCG	
		SOR/LOE	SOR/LOE	Course	SOR/LOE	SOR/LOE	SOR/LOE	Course	SOR/LOE	Course	SOR/LOE
ESMO, 2014 [8]	−/−	−/−	−/−	–	−/−	−/−	−/−	–	−/−	–	−/−
NICE, 2015 [9]^b^	−/−	A/high-low	−/−	At least 6 doses	A/high-low	−/−	−/−	–	−/−	–	−/−
CUA, 2015 [10]	−/−	B/−	B/−	–	−/−	B/−	B/−	1 years	B/−	–	−/−
AUA/SUO, 2016 [3]	Moderate/C	Moderate/B	Moderate/B	6 weeks	Moderate/B	Conditional/C	Moderate/C	–	−/−	1 year	Moderate/C
JUA, 2016 [11]	−/−	−/−	−/−	–	−/−	−/−	−/−	–	−/−	–	−/−
EAU, 2018 [12]	−/−	−/−	Strong/1a-3	–	−/−	Strong/1a-3	Strong/1a-3	≤ 1 years	Weak/3	Full dose, 1 year Three-weekly instillations at 3, 6 and 12 months	Strong/1a-1b
ICUD/SIU, 2018 [13]	A/1a	B/2a	A/1a	–	−/−	B/2a	A/1a	6-12 months	B/2a	Full dose, 1 year	A/1a
CRHA/CPAM, 2018 [14]	B/1a	A/1a	B/1a-1b	Weekly for 4–8 weeks	A/1a	A/1a	A/1a	Monthly for 6–10 months	A/1a	Low dose	B/1a-1b
										1–3 years	B/1b
NCCN, 2019 [15]^c^	−/−	B/2A	A/2A	Initiated 3–4 weeks after TURBT	B/2A	B/2A	A/2A	–	−/−	1 year	B/2A
				Weekly for 6 weeks	B/2A						

^a^ The SOR and LOE are presented as “SOR/LOE”. “-” indicates that the recommendation or evidence was not presented

^b^ To simplify the table, we used “A” and “B” instead of “should/should not/offer/do not offer/refer/advise” or “consider” for presenting SOR

^c^ To simplify the table, we used “A” and “B”, “C” instead of “preferred intervention”, “other recommended intervention”, or “useful in certain circumstances” for presenting SOR

**Table 5:** Recommendations of intravesical therapy for high risk patients^a^

Guideline ID	High risk							
	Induction		Maintenance					
	Be recommended		Be recommended		Course of treatment			
	Chemotherapy	BCG	Chemotherapy	BCG	Chemotherapy		BCG	
	SOR/LOE	SOR/LOE	SOR/LOE	SOR/LOE	Course	SOR/LOE	Course	SOR/LOE
ESMO, 2014 [8]	−/−	−/−	−/−	−/−	–	−/−	–	−/−
NICE, 2015 [9]^b^	−/−	A/high-very low	−/−	A/high-very low	–	−/−	–	−/−
CUA, 2015 [10]	−/−	A/−	−/−	B/−	–	−/−	Full dose, 3 years	B/−
AUA/SUO, 2016 [3]	−/−	Strong/B	−/−	Moderate/B	–	−/−	3 years	Moderate/B
JUA, 2016 [11]	−/−	−/−	−/−	−/−	–	−/−	–	−/−
EAU, 2018 [12]	−/−	−/−	−/−	Strong/1a-1b	–	−/−	Full dose, 1–3 years Three-weekly instillations at 3, 6, 12, 18, 24, 30 and 36 months	Strong/1a-1b
ICUD/SIU, 2018 [13]	B/2	A/1	C/2	B/2	1 year	C/2	3 years	B/2
CRHA/CPAM, 2018 [14]	A/1a	A/1a	A/1a	A/1a	Monthly for 6–10 months	A/1a	Full dose, 3 years	B/1b
NCCN, 2019 [15]^c^	B/2A	A/1	B/2A	A/2A	–	B/2A	3 years Three-weekly instillations at 3, 6, 12, 18, 24, 30 and 36 months	B/2A

^a^ The SOR and LOE are presented as “SOR/LOE”. “-” indicates that the recommendation or evidence was not presented

^b^ To simplify the table, we used “A” and “B” instead of “should/should not/offer/do not offer/refer/advise” or “consider” for presenting SOR

^c^ To simplify the table, we used “A” and “B”, “C” instead of “preferred intervention”, “other recommended intervention”, or “useful in certain circumstances” for presenting SOR

## Discussion

### The rigour of CPG development needs to be improved in the future

The rigour of development could be an important domain for measuring the credibility of guidelines. The most effective CPGs should incorporate the current best evidence and place it in the context of local settings. Failure to use SRs to support their recommendations or to make explicit links between the supporting evidence and the recommendation still existed in some guidelines.

If recommendations were made, the strength is linked directly to the consideration of benefit and harm. Research for intervention safety should be conducted and safety outcomes should be set as key outcomes to balance benefit and harm. A transparent process for reaching consensus is vital for guideline validity, and it is also necessary to record details of all processes by which evidence was appraised and how recommendations were formulated.

### Consumer involvement in cancer-related guidelines

Consumers are broadly defined as recipients of health care who provide a layperson’s perspective and can help in reaching consensus regarding the appropriate rating, presenting recommendations in ways that are understandable to patients and respectful of their needs and acting as a safeguard against conflicts of interests [18].

For example, a patient might consider that the potential benefits in terms of survival might not be worthwhile in view of the potential important, even life-threatening side effects, of a given treatment. Therefore, it is important to consider patient views and expectations in cancer-related treatment recommendations.

BCG instillation has more noticeable side effects than chemotherapy, so the balance between benefit and harm it should be given special attention when making recommendations, especially when attributing the SOR.

### The need to improve the implementation of guidelines during the development process

The score of the applicability domain was disturbingly low, indicating that guideline panels considered the development and implementation of the guidelines as separate activities and did not pay enough attention to the potential facilitators and barriers to the guideline dissemination [19].

To facilitate implementation, guideline panels should consider the publication types and format when reporting the guidelines. Some derivative products were specifically tailored for the target users, including summaries, algorithms and wall charts [20]. Some other resources, such as commissioning support, including audit, measurement and bench marking tools, might be needed as well [16].

Furthermore, disparities in available resources for health care were enormous and shocking. Most included CPGs were developed for situations having full resources so incurring the maximal level of costs, making the applicability of limited utility. Cost-effectiveness analyses were needed for a sensible recommendation especially for developing countries. Economic evaluation should start during scoping of the guidelines. A reliable health economist shall be available to give advice on which questions are likely to require an assessment, and conduct the assessment and then report the results prior to the formulation of recommendations [21].

### Recommendations varied in detail for a variety of reasons

Although most CPGs recommended TURBT and intravesical therapy, they differed in some details such as indications for re-TURBT and the use of chemotherapy agents and BCG in intermediate and high risk NMIBC.

The reasons for offering different recommendations were undoubtedly multifactorial, which might in part be explained by the fact that the guidelines were produced by organizations from different contexts and settings. It could be possible that some discrepancy in guidelines arose through limitations in the current evidence for guideline panels to support their recommendations. In addition, the lack of a transparent process for recommendation formulation resulted in the risk of current evidence having been interpreted differently, because of the different weighting given to certain outcomes during decision making process.

Notably, the recommendations were mostly based on low and moderate quality evidence, whereas the SOR results rated strong plus moderate accounted for a higher proportion. The lack of high-quality evidence might have increased the role that the decision-makers’ opinion had to play in framing the recommendations. Apart from the methodology of guideline development, guideline panels need to focus more on the growing body of evidence.

### Issues that need to be resolved to optimize the treatment

Although the recommendations covered most areas for managing NMIBC patients, some issues that need to be resolved for optimizing treatment have been indicated in some guidelines.

The first important item was whether the second TURBT should be performed after the intravesical therapy followed by the TURBT and whether intravesical therapy should be offered before pathology reports are available. The ESMO guidelines described re-TURBT as a reasonable option in high-risk NMIBC tumours after intravesical therapy, whereas the grade of the recommendation was rated low at III.^8^ The need for further research was obvious.

Such an acknowledged item was which BCG strain is the safest and most effective option [3, 10, 12–14]. Different BCG strains have been implicated in determining responses to BCG, and some strains could influence antitumour immune responses as has been suggested by clinical studies comparing different BCG strains [22]. However, the trial did not reach statistical significance for progression free survival, and none of the CPGs could offer related recommendations. Further evaluation using prospective trials might be needed [12, 23].

Different drug combinations of BCG, chemotherapeutic agents and interferon have been evaluated in various studies, such as interferon plus BCG [24], interferon plus epirubicin [25], BCG plus MMC [26], or BCG plus isoniazid [27]. While CPGs don’t really recommended an optimal combination option, probably because of insufficient evidence, no significant different decrease in recurrence and progression could be found for any of these combination therapies [3, 9, 10, 12, 14].

Despite the disappointing results of combination therapy to date, device-assisted therapies have shown some promising data. Several studies have evaluated the efficacy of hyperthermia to improve the penetration of chemotherapy agents into the bladder wall, thus potentially improving outcomes [28]. The use of electromotive drug administration (EMDA) has been demonstrated to reduce recurrence rates and prolong disease-free intervals [29]. The definitive conclusion, however, needs additional studies to further validate their efficacy as first- and second-line treatments [10, 12].

### Limitations and strengths

Our study might have some potential limitations. First, various grading systems to rate the LOE and SOR make it difficult to compare LOE and SOR among guidelines. Second, recommendations about BCG relapse and radical cystectomy have not been extracted from guidelines, causing the presentation and synthesis of recommendations on the management of NMIBC to be potentially incomplete.

Nonetheless, our present study was reliable and helpful. First, a systematic literature search was conducted for screening eligible CPGs. Second, the reviewers applied AGREE II quality criteria to each CPG and achieved excellent interrater agreement. Furthermore, this is the first attempt to systematically synthesize and appraise CPGs for NMIBC management.

## Conclusions

The quality of NMIBC guidelines in the past 5 years was moderate. The included guidelines often failed to meet the methodological criteria for ideal development and implementation as described by AGREE II. Notwithstanding many consistencies, the recommendations were sometimes inconsistent in details; to what extent this was attributable to the underlying development process remained unclear.

## Supplementary information

**Additional file 1.** Search strategy on PubMed. An exhaustive search was performed in the PubMed using a combination of text-free terms and their corresponding MeSH terms. The search strategy on PubMed is outlined in Additional file 1.

**Additional file 2.** A list of the websites with potential NMIBC guidelines. We searched the websites of guideline development organizations and professional societies. A list of the websites with potential NMIBC guidelines are outlined in Additional file 2.

**Additional file 3.** A composite grading system for ranking evidence and recommendations in NMIBC guidelines. Various grading systems have been used to evaluate the LOE and SOR in different guidelines, for the convenience of statistics, we discussed and reached a consensus on a composite grading system generated as a table in Additional file 3 for presenting the evidence and recommendations.

**Additional file 4.** AGREE II domain score of included CPGs for NMIBC. A bar chart was provided in Additional file 4 in order to present the AGREE II domain score of included CPGs clearly.

**Additional file 5.** Grading systems used and descriptions of evidence and recommendation in the identified guidelines. The grading systems used and descriptions of evidence and recommendation in the identified CPGs were listed in Additional file 5.

**Additional file 6.** Distribution of the SOR and LOE among the identified guidelines on management of NMIBC. A total of 177 recommendations on the management of NMIBC were extracted for statistics. The distribution of the SOR and LOE among those recommendations was displayed in Additional file 6.

**Additional file 7.** Recommendations of immediate postoperative instillation. To demonstrate differences between the identified guidelines, the key recommendations for the management of NMIBC were extracted and summarized. The recommendations of immediate postoperative instillation were synthesized and presented as a table in Additional file 7.

**Additional file 8.** Recommendations of measures for optimizing chemotherapy administration. The recommendations of measures for optimizing chemotherapy administration were synthesized and presented as a table in Additional file 8.

**Additional file 9.** Recommendations of side effects and contraindication of BCG. The recommendations of side effects and contraindication of BCG were synthesized and presented as a table in Additional file 9.

## Data Availability

The datasets generated and/or analysed during the current study are available in the PubMed, Embase, Web of Science and CNKI database. The datasets used and/or analysed during the current study are available from the corresponding author on reasonable request. All data generated or analysed during this study are included in this published article and its additional files.

## Abbreviations

AGREE II: Appraisal of guidelines research & evaluation II
BCG: Bacille Calmette-Guérin
CBM: Chinese biomedical literature database
CI: Confidence interval
CIS: Carcinoma in situ
COI: Conflicts of interest
CPG: Clinical practice guideline
GRADE: The grading of recommendations assessment, development and evaluation
HG: High grade
ICC: Intraclass correlation coefficient
IQR: Interquartile range
LG: Low grade
LOE: Level of evidence
MIBC: Muscle-invasive bladder cancer
MMC: Mitomycin C
NMIBC: Non-muscle-invasive bladder cancer
RCT: Randomized controlled trial
RS: Risk stratification
SOR: Strength of recommendation
SR: Systematic review
TURBT: Transurethral resection of bladder tumour
