# Supplementary material for: Management of non-muscle-invasive bladder cancer: quality of clinical practice guidelines and variations in recommendations
Source: BMC Cancer. 2019 Nov 6;19:1054. doi: 10.1186/s12885-019-6304-y (PMC6836507; doi:10.1186/s12885-019-6304-y)
Supplement: Supplementary file 1 — Additional file 1. Search strategy on PubMed. An exhaustive search was performed in the PubMed using a combination of text-free terms and their corresponding MeSH terms. The search strategy on PubMed is outlined in Additional file 1. [file 12885_2019_6304_MOESM1_ESM.pdf]

#1 non-muscle[Title/Abstract] OR nonmuscle[Title/Abstract] OR non muscle[Title/Abstract] OR without[Title/Abstract]
#2 #1 AND invasive[Title/Abstract]
#3 non-muscle-invasive[Title/Abstract] OR low risk[Title/Abstract] OR superficial[Title/Abstract] OR early[Title/Abstract] OR carcinoma in situ[Title/Abstract] OR Tis[Title/Abstract] OR Ta[Title/Abstract] OR T1[Title/Abstract]
#4 #2 OR #3
#5 Urinary Bladder Neoplasms[Mesh Terms]
#6 Urinary Bladder[Mesh Terms] OR bladder*[Title/Abstract] OR urin*[Title/Abstract] OR urotheli*[Title/Abstract] OR urethra*[Title/Abstract] OR ureter*[Title/Abstract] OR ureteral*[Title/Abstract]
#7 Neoplasms[Mesh Terms] OR cancer*[Title/Abstract] OR tumor*[Title/Abstract] OR tumour*[Title/Abstract] OR neoplas*[Title/Abstract] OR carci*[Title/Abstract] OR malig*[Title/Abstract] OR adenoma*[Title/Abstract] OR adenocarci*[Title/Abstract] OR squamous*[Title/Abstract] OR transitional*[Title/Abstract]
#8 #6 AND #7
#9 #5 OR #8
#10 NMIBC[Title/Abstract] OR non-muscle-invasive bladder cancer[Title/Abstract] OR non-muscle invasive bladder cancer[Title/Abstract] OR Ta bladder cancer[Title/Abstract] OR T1 bladder cancer[Title/Abstract] OR Ta urothelial carcinoma of the urinary bladder[Title/Abstract] OR T1 urothelial carcinoma of the urinary bladder[Title/Abstract] OR Tis bladder cancer[Title/Abstract] OR Tis urothelial carcinoma of the urinary bladder[Title/Abstract] OR superficial bladder carcinoma[Title/Abstract] OR superficial bladder cancer[Title/Abstract]
#11 #4 AND #9
#12 #10 OR #11
#13 Practice Guidelines as Topic[Mesh Terms] OR Practice Guideline[Publication Type] OR Guideline[Publication Type] OR guideline*[Title/Abstract] OR guidance*[Title/Abstract] OR recommendation*[Title/Abstract] OR CPG*[Title/Abstract]
#14 #12 AND #13
