# Supplementary material for: Management of non-muscle-invasive bladder cancer: quality of clinical practice guidelines and variations in recommendations
Source: BMC Cancer. 2019 Nov 6;19:1054. doi: 10.1186/s12885-019-6304-y (PMC6836507; doi:10.1186/s12885-019-6304-y)
Supplement: Supplementary file 2 — Additional file 2. A list of the websites with potential NMIBC guidelines. We searched the websites of guideline development organizations and professional societies. A list of the websites with potential NMIBC guidelines are outlined in Additional file 2. [file 12885_2019_6304_MOESM2_ESM.docx]

Additional file 2 A list of the websites with potential NMIBC guidelines

| **Title** | **Websites** |
| --- | --- |
| Guidelines International Network (GIN) | https://www.g-i-n.net/library/international-guidelines-library/ |
| National Institute for Health and Care Excellence (NICE) | https://www.nice.org.uk/guidance/ng2 |
| Scottish Intercollegiate Guidelines Network (SIGN) | https://www.sign.ac.uk/our-guidelines.html |
| World Health Organization (WHO) | https://www.who.int/ |
| American Society of Clinical Oncology (ASCO) | https://www.asco.org/ |
| American Urological Association (AUA) | https://www.auanet.org/guidelines/bladder-cancer-non-muscle-invasive-(2016)#x2540 |
| European Association of Urology (EAU) | https://uroweb.org/guideline/non-muscle-invasive-bladder-cancer/ |
| National Comprehensive Cancer Network (NCCN) | https://www.nccn.org/professionals/physician_gls/default.aspx |
