# Supplementary material for: Management of non-muscle-invasive bladder cancer: quality of clinical practice guidelines and variations in recommendations
Source: BMC Cancer. 2019 Nov 6;19:1054. doi: 10.1186/s12885-019-6304-y (PMC6836507; doi:10.1186/s12885-019-6304-y)
Supplement: Supplementary file 3 — Additional file 3. A composite grading system for ranking evidence and recommendations in NMIBC guidelines. Various grading systems have been used to evaluate the LOE and SOR in different guidelines, for the convenience of statistics, we discussed and reached a consensus on a composite grading system generated as a table in Additional file 3 for presenting the evidence and recommendations. [file 12885_2019_6304_MOESM3_ESM.docx]

Additional file 3 A composite grading system for ranking evidence and recommendations in NMIBC guidelines

| Category | Grade | Definition |
| --- | --- | --- |
| Level of evidence | 1 | RCTs without important limitations, or SRs of such RCTs |
|  | 2 | RCTs with important limitations, or strong observational studies with consistent findings, or SRs of such studies |
|  | 3 | Nonrandomized studies, cohort or case-control studies, or SRs of such studies |
|  | 4 | Case series, or case reports, or clinical principle/expert opinion |
| Strength of recommendation | A | Strong recommendation with substantial net benefit or harm that can apply to most patients in many circumstances |
|  | B | Moderate recommendation with moderate net benefit or harm that can apply to most patients in many circumstances |
|  | C | Weak or conditional recommendation without apparent net benefit or harm. The best action may differ depending on circumstances or patients’ or societal values |
|  | D | Insufficient evidence on which to formulate a recommendation. The benefit and harm of the treatment are equivalent |

RCT, randomized controlled trial; SR, systematic review.
