# Supplementary figures and images for: Management of non-muscle-invasive bladder cancer: quality of clinical practice guidelines and variations in recommendations
Source: BMC Cancer. 2019 Nov 6;19:1054. doi: 10.1186/s12885-019-6304-y (PMC6836507; doi:10.1186/s12885-019-6304-y)

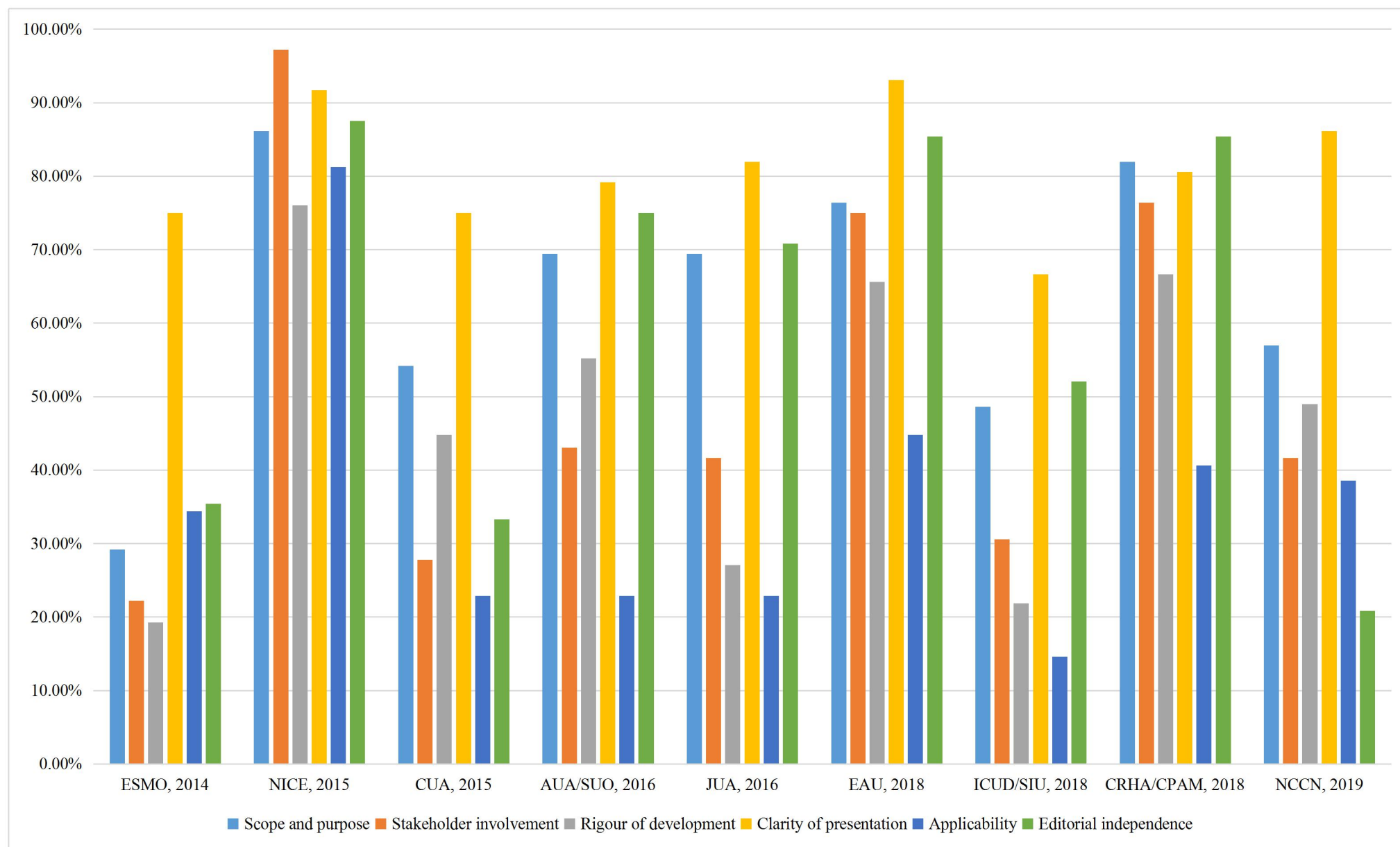

Supplement: Supplementary file 4 — Additional file 4. AGREE II domain score of included CPGs for NMIBC. A bar chart was provided in Additional file 4 in order to present the AGREE II domain score of included CPGs clearly. [file 12885_2019_6304_MOESM4_ESM.pdf]
