# Supplementary material for: Management of non-muscle-invasive bladder cancer: quality of clinical practice guidelines and variations in recommendations
Source: BMC Cancer. 2019 Nov 6;19:1054. doi: 10.1186/s12885-019-6304-y (PMC6836507; doi:10.1186/s12885-019-6304-y)
Supplement: Supplementary file 5 — Additional file 5. Grading systems used and descriptions of evidence and recommendation in the identified guidelines. The grading systems used and descriptions of evidence and recommendation in the identified CPGs were listed in Additional file 5. [file 12885_2019_6304_MOESM5_ESM.docx]

Additional file 5 Grading systems used and descriptions of evidence and recommendation in the identified guidelines

| Guideline ID | Level of Evidence | | Strength of Recommendation | |
| --- | --- | --- | --- | --- |
|  | Grading system used | Description of evidence | Grading system used | Description of recommendation |
| ESMO, 2014 [8] | the Infectious Diseases Society of America-United States Public Health Service Grading System | I, II, III, IV, V | the Infectious Diseases Society of America-United States Public Health Service Grading System | A, B, C, D, E |
| NICE, 2015 [9] | GRADE | High, Moderate, Low, Very low | GRADE | Must/Must not, Should/Should not/Offer/Do not offer/Refer/Advise, Consider |
| CUA, 2015 [10] | OCEBM | 1a, 1b, 1c, 2a, 2b, 2c, 3a, 3b, 4, 5 | OCEBM | A, B, C, D |
| AUA/SUO, 2016 [3] | The AUA categorization of evidence strength | A (high), B (moderate), C (low), Clinical principle/Expert opinion | The AUA nomenclature system | Strong, Moderate, Conditional, Clinical principle/Expert opinion |
| JUA, 2016 [11]^a^ | - | - | - | A, B, C1, C2, D |
| EAU, 2018 [12] | A modified OCEBM | 1a, 1b, 1c, 2a, 2b, 2c, 3a, 3b, 4, 5 | A modified GRADE methodology | Strong, Weak |
| ICUD/SIU, 2018 [13] | OCEBM | 1a, 1b, 1c, 2a, 2b, 2c, 3a, 3b, 4, 5 | OCEBM | A, B, C, D |
| CRHA/CPAM, 2018 [14] | 2015 EAU guidelines standard on levels of evidence | 1a, 1b, 2a, 2b, 3, 4 | 2015 EAU guidelines standard on grading of recommendations | A, B, C |
| NCCN, 2019 [15] | NCCN categories of evidence and consensus | 1, 2A, 2B, 3 | NCCN categories of preference | Preferred intervention, Other recommended intervention, Useful in certain circumstances |

OCEBM, the Oxford Centre for Evidence-based Medicine; [GRADE](http://www.so.com/link?m=aS32PST8icWkDfR0Bo4OcqLLpZoO8oMM1CRBS1kPozVkKAIsyKPDBE0aKJklTSx4ViH15eJgJX/d2PPAbAshtBTglKdA=), the Grading of Recommendations Assessment, Development and Evaluation.

^a^ The SOR set based on agreement among the members of the committee for establishment of Clinical Practice Guidelines for Bladder Cancer.
