# Supplementary material for: Management of non-muscle-invasive bladder cancer: quality of clinical practice guidelines and variations in recommendations
Source: BMC Cancer. 2019 Nov 6;19:1054. doi: 10.1186/s12885-019-6304-y (PMC6836507; doi:10.1186/s12885-019-6304-y)
Supplement: Supplementary file 6 — Additional file 6. Distribution of the SOR and LOE among the identified guidelines on management of NMIBC. A total of 177 recommendations on the management of NMIBC were extracted for statistics. The distribution of the SOR and LOE among those recommendations was displayed in Additional file 6. [file 12885_2019_6304_MOESM6_ESM.docx]

Additional file 6 Distribution of the SOR and LOE among the identified guidelines on management of NMIBC

| Guideline ID | No. of recommendation^a^ | Strength of Recommendation, No. (%) | | | | No. of evidence | Level of Evidence, No. (%) | | | |
| --- | --- | --- | --- | --- | --- | --- | --- | --- | --- | --- |
|  |  | A | B | C | D |  | 1 | 2 | 3 | 4 |
| ESMO, 2014 [8] | 4 | 1(25.0) | 3(75.0) | 0(0) | 0(0) | 4 | 1(25.0) | 1(25.0) | 2(50.0) | 0(0) |
| NICE, 2015 [9] | 14 | 12(85.7) | 2(14.3) | 0(0) | 0(0) | 230 | 15(6.5) | 135(58.7) | 40(17.4) | 40(17.4) |
| CUA, 2015 [10] | 14 | 3(21.4) | 7(50.0) | 3(21.4) | 1(7.1) | 13 | 4(30.8) | 3(23.1) | 6(46.1) | 0(0) |
| AUA/SUO, 2016 [3] | 18 | 3(16.7) | 11(61.1) | 2(11.1) | 2(11.1) | 18 | 0(0) | 6(33.3) | 10(55.6) | 2(11.1) |
| JUA, 2016 [11] | 4 | 1(25.0) | 2(50.0) | 1(25.0) | 0(0) | - | - | - | - | - |
| EAU, 2018 [12] | 22 | 16(72.7) | 0(0) | 6(27.3) | 0(0) | 60 | 30(50.0) | 6(10) | 23(38.3) | 1(1.7) |
| ICUD/SIU, 2018 [13] | 29 | 6(20.7) | 10(34.5) | 11(38.0) | 2(6.9) | 29 | 3(10.3) | 19(65.5) | 7(24.1) | 0(0) |
| CRHA/CPAM, 2018 [14] | 32 | 14(43.8) | 17(53.1) | 1(3.1) | 0(0) | 32 | 20(62.5) | 0(0) | 0(0) | 12(37.5) |
| NCCN, 2019 [15] | 40 | 4(10.0) | 36(90.0) | 0(0) | 0(0) | 40 | 4(10.0) | 35(87.5) | 1(2.5) | 0(0) |
| Total, No. (%) | 177 | 60(33.9) | 88(49.7) | 24(13.6) | 5(2.8) | 426 | 77(18.1) | 205(48.1) | 89(20.9) | 55(12.9) |
