# Supplementary material for: Management of non-muscle-invasive bladder cancer: quality of clinical practice guidelines and variations in recommendations
Source: BMC Cancer. 2019 Nov 6;19:1054. doi: 10.1186/s12885-019-6304-y (PMC6836507; doi:10.1186/s12885-019-6304-y)
Supplement: Supplementary file 7 — Additional file 7. Recommendations of immediate postoperative instillation. To demonstrate differences between the identified guidelines, the key recommendations for the management of NMIBC were extracted and summarized. The recommendations of immediate postoperative instillation were synthesized and presented as a table in Additional file 7. [file 12885_2019_6304_MOESM7_ESM.docx]

Additional file 7 Recommendations of immediate postoperative instillation^a^

| Guideline ID | Immediate postoperative instillation of intravesical chemotherapy | | | | | | | | |
| --- | --- | --- | --- | --- | --- | --- | --- | --- | --- |
|  | Be recommended | | Single dose | Within 24 hours | | Recommended agents | | Contraindication | |
|  | RS | SOR/LOE | SOR/LOE | Supplement | SOR/LOE | Type of agents | SOR/LOE | Description | SOR/LOE |
| ESMO, 2014 [8] | According to risk stratification | A/I | -/- | - | -/- | - | -/- | - | -/- |
| NICE, 2015 [9]^b^ | - | A/high-low | A/high-low | At the same time as the first TURBT | A/high-low | MMC | A/high-low | - | -/- |
| CUA, 2015 [10] | - | B/- | B/- | - | -/- | - | -/- | Bladder perforation or deep/extensive resection | C/- |
| AUA/SUO, 2016 [3] | Low risk, intermediate risk | Moderate/B | Moderate/B | - | Moderate/B | MMC, epirubicin | Moderate/B | Bladder perforation or extensive resection | Moderate/B |
| JUA, 2016 [11] | - | -/- | -/- | - | -/- | - | -/- | -/- |  |
| EAU, 2018 [12] | Low risk, intermediate risk with recurrence rate ≤ 1 per year and EORTC < 5 | Strong/1a-3 | Weak/1a-3 | - | Weak/3 | - | -/- | Bladder perforation or bleeding requiring bladder irrigation | Strong/- |
| ICUD/SIU, 2018 [13] | - | A/1a | A/1a | - | -/- | -/- |  | - | -/- |
| CRHA/CPAM, 2018 [14] | - | B/1a | B/1a | - | -/- | MMC, epirubicin, doxorubicin, pirarubicin, hydroxycamptothecine | B/1a | Bladder perforation or severe haematuria | C/4 |
| NCCN, 2019 [15]^c^ | EORTC < 5, tumors < 8, recurrence rate < 1 per year | B/2A | B/2A | Ideally within 6 hours | B/2A | Gemcitabine | A/1 | Bladder perforation or known drug allergy | B/2A |
|  |  |  |  |  |  | MMC | B/1 |  |  |

RS, risk stratification.

^a^ The SOR and LOE are presented as “SOR/LOE”. “-” indicates that the recommendation or evidence was not presented.

^b^ To simplify the table, we used “A” and “B” instead of “should/should not/offer/do not offer/refer/advise” or “consider” for presenting SOR.

^c^ To simplify the table, we used “A” and “B”, “C” instead of “preferred intervention”, “other recommended intervention”, or “useful in certain circumstances” for presenting SOR.
