# Supplementary material for: Management of non-muscle-invasive bladder cancer: quality of clinical practice guidelines and variations in recommendations
Source: BMC Cancer. 2019 Nov 6;19:1054. doi: 10.1186/s12885-019-6304-y (PMC6836507; doi:10.1186/s12885-019-6304-y)
Supplement: Supplementary file 8 — Additional file 8. Recommendations of measures for optimizing chemotherapy administration. The recommendations of measures for optimizing chemotherapy administration were synthesized and presented as a table in Additional file 8. [file 12885_2019_6304_MOESM8_ESM.docx]

Additional file 8 Recommendations of measures for optimizing chemotherapy administration^a^

| Guideline ID | To optimize chemotherapy administration | | | | | |
| --- | --- | --- | --- | --- | --- | --- |
|  | Reduce fluid intake, alkalinize urine | High concentration | The length of instillation | | Others | |
|  | SOR/LOE | SOR/LOE | Length | SOR/LOE | Description | SOR/LOE |
| ESMO, 2014 [8] | -/- | - | - | -/- | - | -/- |
| NICE, 2015 [9]^b^ | -/- | - | - | -/- | - | -/- |
| CUA, 2015 [10] | B/- | B/- | - | -/- | - | - |
| AUA/SUO, 2016 [3] | -/- | - | - | -/- | - | -/- |
| JUA, 2016 [11] | -/- | - | - | -/- | - | -/- |
| EAU, 2018 [12] | Strong/1b | - | 1-2h | Weak/3 | Control the free flow of the bladder catheter at the end of the immediate instillation | Strong/- |
| ICUD/SIU, 2018 [13] | -/- | - | - | -/- | - | -/- |
| CRHA/CPAM, 2018 [14] | B/1b | B/1a | 0.5-2h | B/1b | High dose | B/1a |
| NCCN, 2019 [15]^c^ | -/- | - | - | -/- | - | -/- |

^a^ The SOR and LOE are presented as “SOR/LOE”. “-” indicates that the recommendation or evidence was not presented.

^b^ To simplify the table, we used “A” and “B” instead of “should/should not/offer/do not offer/refer/advise” or “consider” for presenting SOR.

^c^ To simplify the table, we used “A” and “B”, “C” instead of “preferred intervention”, “other recommended intervention”, or “useful in certain circumstances” for presenting SOR.
