# Supplementary material for: Management of non-muscle-invasive bladder cancer: quality of clinical practice guidelines and variations in recommendations
Source: BMC Cancer. 2019 Nov 6;19:1054. doi: 10.1186/s12885-019-6304-y (PMC6836507; doi:10.1186/s12885-019-6304-y)
Supplement: Supplementary file 9 — Additional file 9. Recommendations of side effects and contraindication of BCG. The recommendations of side effects and contraindication of BCG were synthesized and presented as a table in Additional file 9. [file 12885_2019_6304_MOESM9_ESM.docx]

Additional file 9 Recommendations of side effects and contraindication of BCG^a^

| Guideline ID | BCG side effects/BCG intolerance | | Contraindication of BCG | | | |
| --- | --- | --- | --- | --- | --- | --- |
|  |  |  | Traumatic catheterization, gross hematuria, urinary tract infection | During the first two weeks after TURBT | Others | |
|  | Recommend measures | SOR/LOE | SOR/LOE | SOR/LOE | Description | SOR/LOE |
| ESMO, 2014 [8] | - | -/- | -/- | -/- | - | -/- |
| NICE, 2015 [9]^b^ | Do not offer primary prophylaxis to prevent BCG-related bladder toxicity except as part of a clinical trial | A/high-very low | -/- | -/- | - | -/- |
|  | Seek advice from a specialist urology multidisciplinary team if symptoms of bladder toxicity after BCG cannot be controlled with antispasmodics or non-opiate analgesia and other causes have been excluded by cystoscopy | A/high-very low |  |  |  |  |
| CUA, 2015 [10] | Dose reduction | B/- | -/- | -/- | - | -/- |
| AUA/SUO, 2016 [3] | - | -/- | -/- | -/- | - | -/- |
| JUA, 2016 [11] | - | -/- | -/- | -/- | - | -/- |
| EAU, 2018 [12] | - | -/- | Strong/3 | Strong/- | - | -/- |
| ICUD/SIU, 2018 [13] | Dose reduction | B/2 | -/- | -/- | - | -/- |
| CRHA/CPAM, 2018 [14] | Delay or cessation of therapy | A/4 | A/4 | A/4 | Severe immunosuppression, active pulmonary tuberculosis | A/4 |
| NCCN, 2019 [15]^c^ | Dose reduction | B/2A | B/2A | -/- | Severe local symptoms, or systemic symptoms | B/2A |

^a^ The SOR and LOE are presented as “SOR/LOE”. “-” indicates that the recommendation or evidence was not presented.

^b^ To simplify the table, we used “A” and “B” instead of “should/should not/offer/do not offer/refer/advise” or “consider” for presenting SOR.

^c^ To simplify the table, we used “A” and “B”, “C” instead of “preferred intervention”, “other recommended intervention”, or “useful in certain circumstances” for presenting SOR.
